# Transcriptional Control and mRNA Capping by the GDP Polyribonucleotidyltransferase Domain of the Rabies Virus Large Protein

**DOI:** 10.3390/v11060504

**Published:** 2019-06-01

**Authors:** Tomoaki Ogino, Todd J. Green

**Affiliations:** 1 Department of Molecular Biology and Microbiology, Case Western Reserve University School of Medicine, Cleveland, OH 44106, USA; 2 Department of Inflammation and Immunity, Lerner Research Institute, Cleveland Clinic, Cleveland, OH 44195, USA; 3 Department of Microbiology, School of Medicine, University of Alabama at Birmingham, Birmingham, AL 35294, USA; tgreen@uab.edu

**Keywords:** rabies virus, L protein, transcription, mRNA capping, GDP polyribonucleotidyltransferase, de novo initiation

## Abstract

Rabies virus (RABV) is a causative agent of a fatal neurological disease in humans and animals. The large (L) protein of RABV is a multifunctional RNA-dependent RNA polymerase, which is one of the most attractive targets for developing antiviral agents. A remarkable homology of the RABV L protein to a counterpart in vesicular stomatitis virus, a well-characterized rhabdovirus, suggests that it catalyzes mRNA processing reactions, such as 5′-capping, cap methylation, and 3′-polyadenylation, in addition to RNA synthesis. Recent breakthroughs in developing in vitro RNA synthesis and capping systems with a recombinant form of the RABV L protein have led to significant progress in our understanding of the molecular mechanisms of RABV RNA biogenesis. This review summarizes functions of RABV replication proteins in transcription and replication, and highlights new insights into roles of an unconventional mRNA capping enzyme, namely GDP polyribonucleotidyltransferase, domain of the RABV L protein in mRNA capping and transcription initiation.

## 1. Introduction

Rabies virus (RABV) is a nonsegmented negative-strand (NNS) RNA virus belonging to the *Lyssavirus* genus of the *Rhabdoviridae* family in the order *Mononegavirales* (reviewed in References [1,2,3]). RABV is transmitted to humans from infected animals, mainly domestic dogs, through their saliva by biting or scratching, and causes an acute, fatal neurological disease, called rabies [2,3,4]. Although rabies is preventable by vaccination prior to or immediately after exposure, there are currently no established therapeutic countermeasures once patients are symptomatic [4]. RABV kills more than 50,000 people each year worldwide [5,6], posing continuing threats to human health, especially in developing countries with lower vaccination coverage in domestic dogs. In addition, rabies-like diseases are known to occur in humans by infection with other lyssaviruses, such as Duvenhage virus, European bat lyssaviruses 1 and 2, Australian bat lyssavirus, Mokola virus, and Irkut virus, although in rare cases [3,7]. Therefore, it is important to understand the basic biology of lyssaviruses, which can aid in development of therapeutic targets against them. 

Studies on transcription and replication of rhabdoviruses have been carried out mainly using vesicular stomatitis virus (VSV, an arthropod-borne animal vesiculovirus) as a model (reviewed in Reference [8]), because VSV can be safely handled and shows the strongest RNA synthesis activity among rhabdoviruses as well as other NNS RNA viruses belonging to different families. Since purified RABV particles exhibited significantly lower RNA synthesis activities than that of VSV [9,10,11], it has remained difficult to biochemically characterize virion-associated enzymes involved in RNA biosynthesis. Recent advantages in establishing in vitro RNA synthesis and capping assays for RABV have enabled us to elucidate the molecular mechanisms of viral RNA biosynthesis. In this review, we discuss roles of RABV replication proteins in transcription and replication, and focus on recent studies regarding unique RABV machineries required for mRNA capping and transcription initiation.

## 2. Transcription and Replication of the Rabies Virus (RABV) Genome

**Figure 1:**
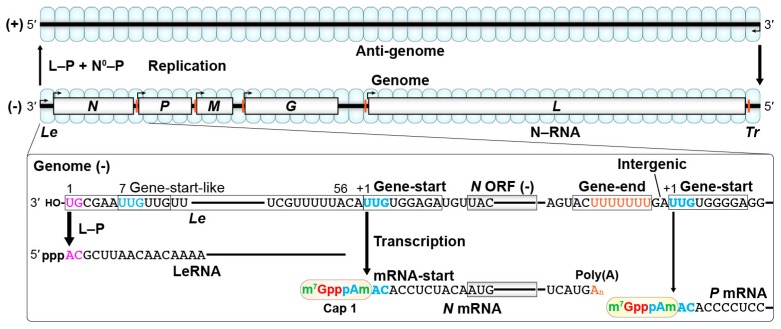
Transcription and replication of the rabies virus (RABV) genome. The negative-strand RABV genome wrapped with the nucleocapsid (N) proteins serves as a template for transcription (lower) and replication (upper). The RNA-dependent RNA polymerase (RdRp, the complex between large (L) and phospho- (P) proteins) sequentially transcribes the leader region (*Le*) and five internal genes (*N*, *P*, *M*, *G*, and *L*) in the genome into the leader RNA (LeRNA) and five monocistronic mRNAs with the 5′-cap 1 and 3′-poly(A) structures by the stop-start transcription mechanism. The RdRp carries out encapsidation-coupled genome replication together with the complex of the RNA-free N protein with the P protein (N^0^-P). Partial RNA sequences of the genome and transcripts of RABV (PV strain, GenBank id: M13215) are shown. The conserved gene-start and gene-end sequences serve as transcription initiation and polyadenylation/termination signals, respectively. The conserved mRNA-start sequence acts as an mRNA capping signal. HO- and ppp indicate hydroxyl and triphosphate groups, respectively. m^7^GpppAm indicates *N*^7^-methylguanosine(5′)triphospho(5′)2′-*O*-methyladenosine (cap 1).

**Figure 2:**
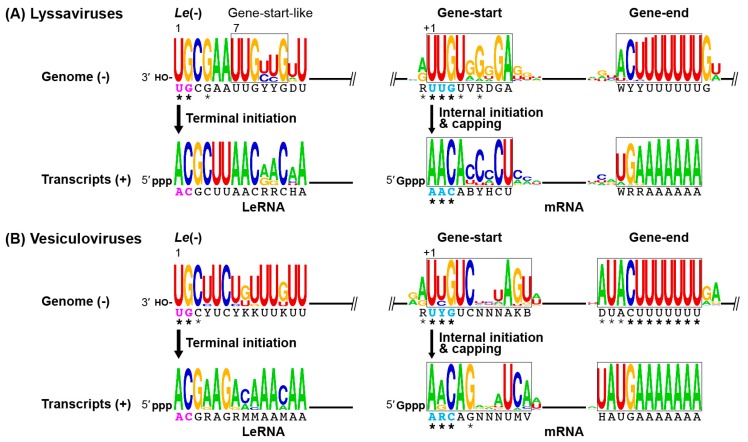
Conserved elements in lyssaviral and vesiculoviral RNAs. The 3′-terminal *Le*(−) promoter (residues 1–14), internal gene-start and -end (from *N*, *P*, *M*, *G*, and *L* genes), 5′-terminal LeRNA (residues 1–14), and mRNA-start and -end (from *N*, *P*, *M*, *G*, and *L* mRNAs) sequences were analyzed by the WebLogo program [39] for lyssaviruses (**A**) rabies virus (RABV) (GenBank id: M13215), Aravan virus (EF614259), Australian bat lyssavirus (AF418014), Bokeloh bat lyssavirus (JF311903), Duvenhage virus (EU293120), European bat lyssavirus 1 (EF157976), European bat lyssavirus 2 (EF157977), Gannoruwa bat lyssavirus (KU244266), Ikoma lyssavirus (JX193798), Irkut virus (EF614260), Khujand virus (EF614261), Lagos bat virus (EU293110), Lleida bat lyssavirus (KY006983), Mokola virus (Y09762), Shimoni bat virus (GU170201), West Caucasian bat virus (EF614258)) and vesiculoviruses (**B**) VSV (vesicular stomatitis Indiana virus, AF473864), vesicular stomatitis Alagoas virus (EU373658), American bat vesiculovirus (JX569193^†^), Carajas virus (KM205015^†^), Chandipura virus (GU212856), Cocal virus (EU373657), Isfahan virus (AJ810084), Jurona virus (KM204996^†^), Malpais Spring virus (KC412247), Maraba virus (HQ660076), Morreton virus (KM205007^†^), vesicular stomatitis New Jersey virus (JX121110), Perinet virus (HM566195^†^), Piry virus (KU178986^†^), Radi virus (KM205024^†^), Yug Bogdanovac virus (JF911700); †, accurate terminal sequences are not available). Consensus sequences are shown under the logos. R is A or G; Y is U or C; W is A or U; K is G or U; M is A or C; B is C, G, or U; D is A, G, or U; H is A, C, or U; V is A, C, or G; N is any nucleotide. The RABV and VSV residues essential and important for transcription initiation or capping are marked by bold and regular asterisks, respectively [21,22,23,40,41,42,43,44,45,46].

The RABV genome of approximately 11.9 kilo nucleotides (nt) is composed of five genes encoding nucleocapsid (N), phospho- (P), matrix (M), glyco- (G), and large (L) proteins [12,13,14,15] that are preceded and followed by the noncoding 3′-leader (*Le*, 58 nt) and 5′-trailer (*Tr*, 70 nt) regions, respectively (Figure 1). Other lyssaviruses possess the same genomic organization as RABV [16,17]. The negative-strand *Le* (*Le*(−)) region on the genome and positive-strand *Tr* (*Tr*(+)) region on the anti-genome contain promoter elements required for synthesis of the anti-genome and genome, respectively [18,19,20,21]. The difference in promoter strength between *Le*(−) and *Tr*(+) determines the molar ratio of the genome to anti-genome of 49:1 in infected cells, eventually leading to packaging of the genome into virus particles more efficiently than the anti-genome [20]. Each lyssaviral gene begins and ends with conserved gene-start and gene-end sequences, respectively (Figure 2A), which may serve as signals for transcription initiation and polyadenylation/termination, respectively, as reported for VSV [22,23]. These lyssaviral transcriptional signals show strong sequence similarities to those of vesiculoviruses (Figure 2B). The genome is encapsidated with the N proteins to form a helical N–RNA complex [24], which acts as a template for transcription and replication. As demonstrated for VSV [25,26,27,28,29], the RABV RNA-dependent RNA polymerase (RdRp) complex composed of the catalytic L and cofactor P proteins is associated with the N–RNA complex to form a transcriptionally active ribonucleoprotein (RNP) complex [30,31,32,33,34]. In host cells, RABV forms cytoplasmic inclusion bodies, similar to Negri Bodies, as liquid-like replication organelles, where viral RNA synthesis as well as RNP assembly takes place [35,36,37]. Similar liquid-like inclusion bodies formed in the cytoplasm of VSV-infected cells were suggested to serve as VSV replication sites [38].

According to the “single entry, stop-start transcription” model proposed for VSV [47,48,49,50,51], the RABV RdRp complex initiates transcription at the 3′-terminal of the *Le*(−) promoter to synthesize the leader RNA (LeRNA), and then sequentially transcribes the five internal genes into monocistronic mRNAs (Figure 1, lower) [52,53,54]. The RABV mRNAs synthesized in infected cells have a 3′-poly(A) tail of 100–250 nt [54], whereas there is no information on modifications to their 5′-ends in infected cells. By analogy to VSV [47,55,56], the 5′-end of the RABV mRNAs is proposed to be modified into methylated cap structures, such as m^7^G(5′)ppp(5′)Am- (cap 1), m^7^G(5′)ppp(5′)AmpAm- (cap 2), and m^7^G(5′)ppp(5′)m^6^Ampm^6^Am- (m^7^G, *N*^7^-methylguanosine; Am, 2′-*O*-adenosine; m^6^Am, *N*^6^,2′-*O*-dimethyladenosine). In RABV-infected cells, major (55 or 56 nt) and minor (57 or 58 nt) forms of LeRNA are synthesized and present as RNPs containing a cellular RNA binding protein, La [57]. As in the case of VSV [48,49,58], stop-start transcription, which is accompanied with attenuation of reinitiation at each gene junction, by the RABV RdRp generates a gradient in mRNA abundance in the following order: *N* > *P* > *M* > *G* > *L* [52,59]. The RABV genes are tandemly connected via intergenic regions (IGRs) of variable lengths (2–24 nt) [13,15], in which longer IGRs (*G*/*L*, 24 nt > *P*/*M* and *M*/*G*, 5 nt > *N*/*P*, 2 nt) more significantly attenuate transcription of downstream genes [59]. The RABV RdRp seems to scan an IGR until a downstream gene-start sequence is encountered after releasing a polyadenylated upstream mRNA. Thus, increasing the length of the IGRs may further decrease the efficiency of scanning for a downstream gene-start sequence, providing an additional mechanism to downregulate reinitiation of downstream transcription. In contrast, VSV (Indiana) possesses IGRs of a fixed length (2 nt) in the genome [60].

For genome replication, the RABV RdRp ignores the signals for mRNA synthesis on the genome to copy it into the positive-strand anti-genome, which is in turn used as a template for synthesis of the genome (Figure 1, upper). The genome and anti-genome are each co-replicationally encapsidated with the N proteins to form the N–RNA templates. As proposed for VSV [61,62,63,64], a complex of an RNA-free N (called N^0^) protein with the P protein, accumulated in RABV-infected cells, may play an essential role in co-replicational nucleocapsid assembly. An N-terminal portion (residues 4–40) of the RABV P protein interacts with the N protein, keeping it in an RNA-free soluble form [65]. Thus, it can be suggested that the N-terminal N^0^-binding domain of the P protein is required for its chaperoning activity that delivers the N^0^ protein to the replication products. For VSV, selective encapsidation of LeRNA with the N proteins was suggested to trigger a mode switch from transcription to replication, leading to encapsidation-coupled elongation of LeRNA to the full-length anti-genome [66,67]. The RABV N protein interacts with an A-rich sequence (residues 20–30) in LeRNA more selectively than with unrelated RNAs in the presence of the P protein [68], suggesting that the selective LeRNA encapsidation is carried out with the N^0^–P complex and thereby leads to genome replication. In addition, the RABV M protein inhibits transcription, but rather stimulates replication, suggesting that the M protein regulates mode switching between transcription to replication [69,70].

## 3. Rabies Virus (RABV) Replication Proteins

**Figure 3:**
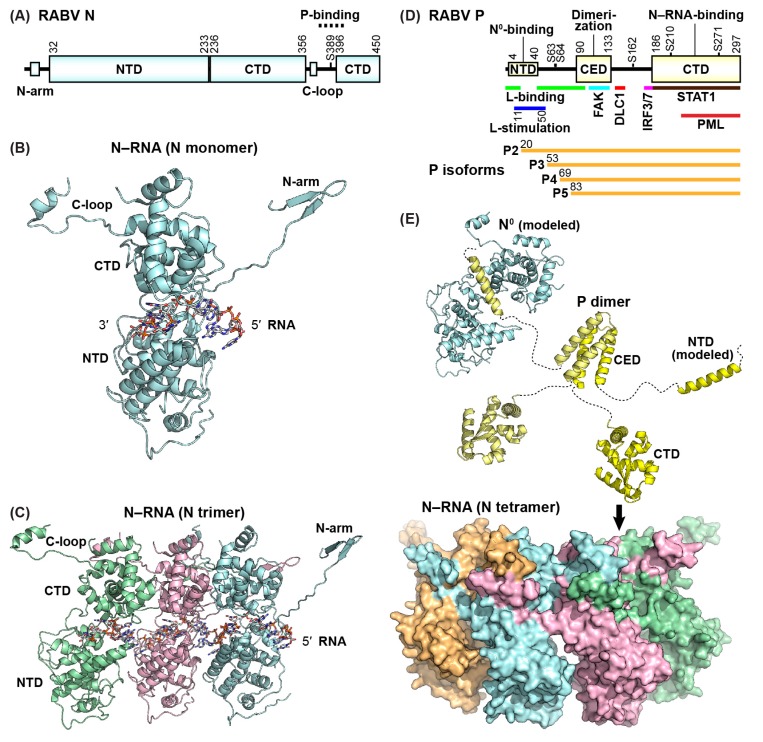
Structures of the rabies virus (RABV) nucleocapsid (N) and phospho- (P) proteins. (**A**) The domain organization of the RABV N protein is schematically shown. NTD and CTD denote N- and C-terminal domains, respectively. Numbers indicates the positions of amino acid residues beginning and ending the domains. A predicted P-binding region on the N–RNA complex is shown by a dashed line. S389 is a phosphorylation site. (**B**) A cartoon representation of the monomeric N protein (PDB id: 2GTT) is shown with encapsidated nine-mer of RNA. Regional landmarks are labeled. (**C**) An assembled trimer of N proteins is shown bound to a 27-mer of RNA. Each monomer of N is shaded in a different color. (**D**) The domain organization of the RABV P protein is depicted with binding sites for RABV (N^0^, RNA-free N protein; L, large protein; N–RNA) and host (FAK, focal adhesion kinase; DLC1, dynein light chain 1; STAT1, signal transducer and activator of transcription 1; PML, promyelocytic leukemia) factors. NTD, CED, and CTD indicate N-terminal, central, and C-terminal domains, respectively. Regions required for stimulation of the L RNA-dependent RNA polymerase activity and inhibition of IRF3/7 (interferon regulatory factor 3/7) activation are shown. Five serine residues (S63, S64, S162, S210, and S271) are noted as phosphorylation sites. P2–5 indicate N-terminal truncation isoforms of the P protein. (**E**) The dimeric RABV P is shown in cartoon form (individual monomers colored yellow and pale yellow) with regional landmarks labeled as in (D). P binds the unassembled N^0^ (top) via the N-terminal region of P. The dimerization domain (PDB id: 3L32) is shown central to the figure. A tetramer of N proteins (shaded in different colors) is shown in surface representation, 180 degrees rotated with a slight tilt forward from the view in (**B**) and (**C**). The C-terminal domain of P (PDB id: 1VYI) binds a bipartite binding site involving the C-loops and the surface on CTD of N (below the arrow) on the assembled nucleocapsid. Linker segments are illustrated as dashed lines. The RABV N^0^–P complex is modeled based on the equivalent vesicular stomatitis virus complex in [78].

The RABV N protein (450 amino acids, Figure 3A) encapsidates the genome and antigenome, to generate the competent templates for RNA synthesis and to protect them from cellular ribonucleases. Recombinant rhabdoviral N proteins are known to be assembled with cellular RNAs into closed ring-like structures containing 10 ± 1 N subunits as well as helical nucleocapsid-like structures, when expressed in insect cells [24,31] or *E. coli* [71,72]. X-ray crystallographic analyses of the ring-like N–RNA complexes of RABV and VSV revealed that the N protein is composed of N- and C-terminal domains, which are oriented in an angled conformation to form an RNA-binding groove (Figure 3B) [72,73]. In the N–RNA complexes, each N subunit is associated with neighboring N subunits using unique N-terminal arm-like and C-terminal loop structures extended from the N- and C-terminal domains, respectively (Figure 3C) [72,73]. Both the N-arm and C-loop structures of the VSV N protein are required for its oligomerization and RNA encapsidation activities [74]. Nine nucleotides of RNA are covered within an RNA-binding groove in each N subunit, in which basic amino acid residues are associated with the RNA phosphate backbone [72,73]. Transcription and replication of a VSV mini-replicon were shown to be abolished or diminished by mutations of the phosphate-binding basic amino acid residues [75]. The C-loop of two adjacent N subunits in the VSV N–RNA complex provides a binding site for the C-terminal domain of the P protein [76]. A similar mode of binding of the C-terminal domain of the RABV P protein to the N–RNA template was proposed from molecular modeling studies [33]. Phosphorylation of the RABV N protein at a serine residue position 389 (S389) is required for efficient transcription and replication [77].

The RABV P protein (297 amino acids, Figure 3D) is an elongated dimeric protein with structured and unstructured regions and is phosphorylated with cellular kinases [79,80,81]. The P protein plays multiple roles during the viral life cycle through direct interactions with various viral and host proteins. The N-terminal (residues 4–40) and C-terminal (residues 186–297) domains of the RABV P protein interacts with the N^0^ protein [65,82] and N–RNA complex [32,33], respectively (Figure 3E). For VSV, the N-terminal N^0^-binding site of the P protein binds adjacent to the RNA-binding groove of the N^0^-protein and a binding site for the N-arm of an adjacent N subunit [78], thereby preventing the nucleocapsid formation. Two independent regions (residues 1–19 and 40–100) of the RABV P protein were reported to bind to a C-terminal part of the L protein [30,34], while its activity that stimulates the RdRp activity of the L protein resides within residues 11–50 [83]. The dimerization domain of the RABV P protein is located in a central region (residues 90–133) [84], but residues 65–175, including the dimerization domain, are dispensable for transcription of an RABV mini-genome in cultured cells [85]. The RABV P protein contains a binding site (residues 139–151) for cellular dynein light chain 1 (DLC1, also called dynein light chain LC8) [86,87,88], which is required for efficient genome transcription rather than originally proposed retrograde axonal transport of RABV in neuronal cells [89]. Cellular focal adhesion kinase also interacts within the central dimerization domain (residues 106–131) of the RABV P protein, and positively regulates the RABV RNA synthesis activity [90]. In addition, the RABV P protein and its N-terminal truncation isoforms (called P2–P5) are known to counteract functions of various cellular factors involved in antiviral responses, such as IRF-3, IRF-7, STAT1, and PML (reviewed in [91]). Residues 176–186 of the RABV P protein are required for inhibition of IRF3/7 activation [92]. The C-terminal domain of the RABV P protein includes binding sites for STAT1 (residues 187–297) [92,93,94] and PML (residues 223–297) [95].

**Figure 4:**
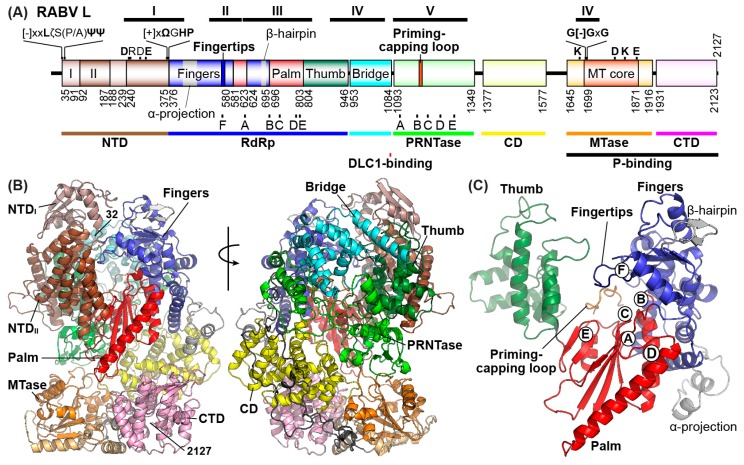
A structural model of the rabies virus (RABV) large (L) protein. (**A**) The domain organization of the RABV L protein was predicted as reported for the vesicular stomatitis virus (VSV) L protein [8]. The predicted domains and subdomains are colored as follows: NTD, N-terminal domain (subdomains: I, light brown; II, brown); RdRp, RNA-dependent RNA polymerase (subdomains: fingers, blue; palm, red; thumb, dark green); Bridge (cyan); PRNTase, GDP polyribonucleotidyltransferase (green); CD, connecter domain (yellow); MTase, methyltransferase (MTase core, orange; other regions, light orange); CTD, C-terminal domain (pink); linker regions between domains (black). Numbers indicates the positions of amino acid residues beginning and ending the domains and subdomains. The positions of the conserved regions (I–VI) [105], RdRp motifs (A–F), PRNTase motifs (A–E), and other motifs are shown [8]. [−], [+], ζ, Ψ, Ω, and x indicate negatively charged, positively charged, hydrophilic, aliphatic, aromatic, and any amino acids, respectively. Binding sites for the RABV P protein and host DLC1 are indicated below the schematic. (**B**) A three-dimensional structure of the RABV L protein (RC-HL strain, GenBank id: AB009663) was predicted with SWISS-MODEL [122] using the structure of the VSV L protein (PDB id: 5A22) as a template. Two views of the structure are shown as ribbon models, in which domains and subdomains are colored as in (**A**). The extended linkers between the PRNTase/CD and CD/MTase domains are visible and noted in in the model to the right. The linkers were not modeled in the previous VSV structure. Amino acid residues at positions 32 and 2127 (C-terminal end) are indicated. N-terminal residues 1–31 are not present in the model. (**C**) The predicted structure of the RABV RdRp domain is shown. The positions of RdRp motifs A–F are denoted by circled capital letters. The priming-capping loop extended from the PRNTase domain is shown in orange. Rhabdovirus-specific substructures in the fingers subdomain are colored gray. All structural images were prepared with PyMOL [123]. The coordinates of the modeled RABV L protein have been uploaded to the Model Archive (www.modelarchive.org, id: ma-pe4nf).

The RABV L protein (2127 amino acids, Figure 4A) is a multi-domain protein that may catalyze all enzymatic reactions required for RNA synthesis and processing (mRNA 5′-capping, cap methylation, and 3′-polyadenylation) as reported for other NNS RNA viral L proteins [43,45,96,97,98,99,100,101,102,103]. At present, for RABV, only RNA synthesis and capping activities have been experimentally demonstrated using recombinant forms of the RABV L protein [21,46,83]. The RABV P protein interacts with the C-terminal part of the L protein [30,34,104], though its precise binding site is still not known. The RABV P protein stimulates transcription initiation [21] as well as elongation [83] mediated by the L protein. The RABV L protein exhibits significant similarities to the well-characterized VSV L protein throughout the entire protein at the amino acid sequence level [105,106,107], suggesting that it has the same domain organization as well as enzymatic functions as the VSV L protein. Since information on a three-dimensional structure of the RABV L protein is not available, its structural model was generated using the structure of the VSV L protein solved by cryo-electron microscopy (PDB id: 5A22) [108] as a template (Figure 4B). As suggested for the VSV L protein [8,108,109], the RABV L protein was predicted to have an N-terminal (NTD, composed of subdomains I and II), RdRp, bridge, mRNA capping enzyme (GDP polyribonucleotidyltransferase, PRNTase), connector (CD), methyltransferase (MTase), and C-terminal (CTD) domains. The RdRp domain of the RABV L protein as well as the VSV L protein [108] is composed of putative fingers, palm, and thumb subdomains, and contains structural motifs A–F for RdRps [8,105,110,111,112]. The palm subdomain of the RABV L protein possesses two universally conserved aspartate residues in motifs A (D618) and C (D729), which may serve as catalytic residues for two-metal dependent nucleotide polymerization as proposed for other polymerases [113,114,115,116,117,118,119]. As predicted, D729 of the RABV L protein was shown to be required for its RNA synthesis activity in vitro [21] as well as in cellula [120]. Interestingly, residues 1079 to 1083 of the RABV L protein interact with cellular DLC1, which stimulates RABV transcription in infected cells and recruits the L protein to reorganized microtubules when overexpressed in transfected cells [121]. However, the degree of microtubule-reorganization with the RABV L protein in infected cells and its precise role(s) in viral replication and pathogenesis have not been studied.

## 4. mRNA Capping in Eukaryotes and Rhabdoviruses

**Figure 5:**
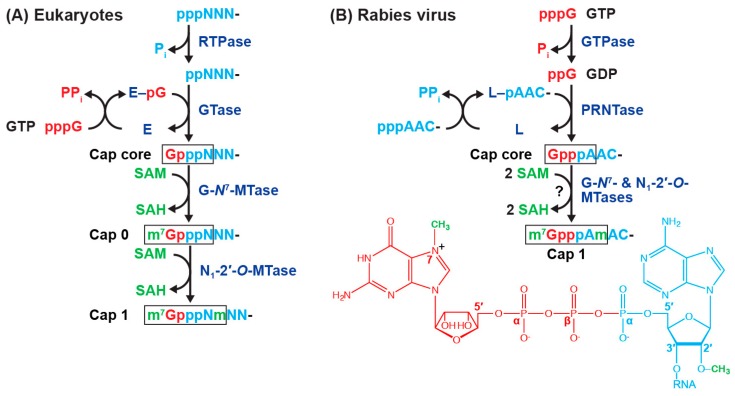
The mechanisms of mRNA capping. Pathways of the mRNA cap formation in higher eukaryotes (**A**) and rabies virus (RABV) (**B**) are represented. RNA (pppNNN-/AAC-), GTP (pppG), and *S*-adenosyl-L-methionine (SAM) are shown in light blue, red, and green, respectively. P_i_, PP_i_, and SAH indicate inorganic phosphate, inorganic pyrophosphate, and *S*-adenosyl-L-homocysteine, respectively. Enzymes are: RTPase, RNA 5′-triphosphatase; GTase, mRNA guanylyltransferase (shown by “E”); GTPase, guanosine 5′-triphosphatase; PRNTase, GDP polyribonucleotidyltransferase (shown by “L”); G-*N*^7^-MTase, mRNA (guanine-*N*^7^)-methyltransferase; N_1_-2′-*O*-MTases, mRNA (nucleoside_1_-2′-*O*-)-methyltransferase. Note that two methylation reactions by RABV enzymes have not been demonstrated. In (**B**, bottom), parts of the cap 1 structure formed with the RABV enzymes are colored differently based on their origins (GTP, RNA, and SAM).

The eukaryotic mRNA cap is a unique 5′-terminal block structure, in which m^7^G is linked to the first nucleoside (N_1_) of mRNA through an inverted 5′-5′ triphosphate bridge (reviewed in [124,125,126,127,128]). In eukaryotic cells, the cap 0 structure (m^7^GpppN_1_-) is formed on pre-mRNA with sequential enzymatic reactions coupled with mRNA chain elongation by DNA-dependent RNA polymerase II (pol II) in the nucleus (Figure 5A) [125,126,127,128]. The cap 0 structure is essential for mRNA biosynthesis and metabolism at various steps, such as mRNA splicing, export, translation, and degradation [124,125,128]. Eukaryotic mRNA capping enzymes are associated with the largest subunit of pol II in an early stage of transcription and carry out co-transcriptional mRNA capping with their RNA 5′-triphosphatase (RTPase) and mRNA guanylyltransferase (GTase) activities [125,126,127,128]. The RTPase activity removes the γ-phosphate group from 5′-triphosphate-ended RNA (pppRNA), producing 5′-diphosphate-ended RNA (ppRNA) and inorganic phosphate (P_i_). The GTase activity subsequently transfers the GMP moiety from GTP to ppN_1_-RNA via a covalent enzyme-(lysyl-*N*^ζ^)–GMP (E–pG) intermediate to generate capped RNA (GpppN_1_-RNA). Then, mRNA (guanine-*N*^7^)-methyltransferase (G-*N*^7^-MTase) transfers a methyl group from *S*-adenosyl-L-methionine (SAM) to the guanine-*N*^7^ position of GpppN_1_-RNA to form m^7^GpppN_1_-RNA (cap 0-RNA). In multicellular eukaryotes, m^7^GpppN_1_-RNA is further methylated by mRNA (nucleoside-2′-*O*-)-methyltransferases (N-2′-*O*-MTases) to yield m^7^GpppN_1_m-RNA (cap 1-RNA) and m^7^GpppN_1_mpN_2_m-RNA (cap 2-RNA) [124]. Importantly, N_1_-2′-*O*-methylation of the cap structure is required for avoiding anti-viral innate immune reactions (reviewed in [129,130]). In higher eukaryotic cells, cap 0-mRNAs are sensed as non-self RNAs by viral RNA sensors, such as RIG-I [131,132] and MDA5 [133], resulting in production of interferon followed by anti-viral factors. The cap 0 structure on viral mRNAs is further recognized by the interferon-inducible IFIT1 protein together with its related proteins to block their translation [134,135,136,137,138,139]. In addition, when N_1_ is adenosine, the cap structure is often methylated at the adenine-*N*^6^ position [140,141].

The unconventional mechanism of rhabdoviral mRNA capping, discovered in vesiculoviruses, such as VSV [43,47,142] and Chandipura virus [143], is strikingly different from the conventional mechanism of eukaryotic mRNA capping (reviewed in [107,144]). In the first step of the VSV capping reaction, GTP is hydrolyzed into GDP by a guanosine 5′-triphosphatase (GTPase) activity associated with the VSV L protein [43,44]. In the second step, the PRNTase domain in the VSV L protein transfers 5′-monophosphate-ended RNA (pRNA) from pppRNA (pRNA donor) to GDP (pRNA acceptor) through a covalent enzyme-(histidyl-*N*^ε^)–pRNA (called L–pRNA) intermediate to generate GpppRNA [43,145]. The VSV L protein specifically recognizes pppRNA, but not ppRNA, with the vesiculoviral mRNA start-sequence, 5′-A_1_R_2_C_3_N_4_G_5_ (R: A or G; N: any nucleotides; the subscript numbers indicate the positions of the nucleotide residues from the 5′-end) (Figure 2B), in which A_1_R_2_C_3_ and G_5_ are essential and important, respectively, for the pRNA transfer reaction at the step of the L–pRNA intermediate formation [43,44,146]. In contrast, the VSV L protein is not able to form an L–pRNA intermediate with the VSV LeRNA start-sequence, 5′-ACGAA [146], explaining why the VSV L protein caps VSV mRNAs but not LeRNA [43,47,142,147]. The PRNTase domain of the VSV L protein employs GDP, but not the other three NDPs, as the pRNA acceptor [145]. The pRNA acceptor activity of GDP requires the *C*^2^-amino group of guanine and 2′ or 3′-hydroxyl group of ribose, but not *C*^6^-oxo group, *N*^1^-hydrogen, or *N*^7^-nitrogen [148]. The PRNTase domain also accepts GTP to generate a tetraphosphate containing cap, G(5′)pppp(5′)A, although to a lesser extent than GDP, when GTP hydrolysis is a rate-limiting step in capping under in vitro conditions [44]. However, the GDP production step appears to be omitted for the GpppA formation in infected cells, because intracellular concentrations of GDP are usually 3–4 orders of magnitude higher than the *K*_m_ for the pRNA acceptor, GDP (0.03 µM) [148]. 

Although there is no direct evidence, the 5′-ends of the RABV mRNAs are thought to be capped and methylated into the cap 1 structure (m^7^GpppAm-) and/or more extensively methylated forms (e.g., m^7^G(5′)ppp(5′)AmpAm-, m^7^G(5′)ppp(5′)m^6^Ampm^6^Am-) in the cytoplasm of infected cells as reported for the VSV mRNAs [47,55,56]. However, it remains challenging to analyze cap structures on RABV mRNAs synthesized either in vitro or in cellula due to their very low quantities [9,10,11]. The recent development of the methods for expression and purification of an enzymatically active recombinant form of the RABV L protein allowed us to demonstrate that it shows GTPase and PRNTase activities to generate the cap structure in vitro [46] (Figure 5B). The latter activity caps pppRNA, but not ppRNA, with GDP in a sequence-dependent manner [46]. As shown in Figure 2A, all the known lyssaviral mRNAs begin with the conserved 5′-A_1_A_2_C_3_A_4_B_5_ (B: C, U, or G). Consistently, the RABV L protein employs pppRNAs with the lyssaviral mRNA start-sequences (e.g., AACAC, AACAU), but not with the LeRNA-start sequence (ACGCU), as pRNA donor substrates [46]. The RABV L protein strictly recognizes the first three nucleotide, A_1_A_2_C_3_, of the pRNA donors, in which A_2_ cannot be replaced with G [46], manifesting its specificity for lyssaviral mRNAs slightly different from that of the VSV L protein [43,44]. Since the PRNTase domain of the RABV L protein exhibits a very low specific capping activity approximately 600-fold lower than that of the VSV L protein [46], it remains particularly difficult to demonstrate the formation of the putative RABV L–pRNA intermediate.

On the other hand, it is not known whether the putative MTase domain in the RABV L protein catalyzes cap methylation. The single cap MTase domain of the VSV L protein was suggested to catalyze sequential N_1_-2′-*O*- and G-*N*^7^-methylation reactions (GpppA- → GpppAm- → m^7^GpppAm-) in an opposite way to those by eukaryotic cap MTases [97,98,101,149,150,151]. The putative RABV MTase domain was predicted to have a SAM-dependent MTase core fold (Figure 4) similar to that of the VSV L protein [108] and contains a glycine-rich SAM binding motif G[−]GxG ([−], negatively charged amino acids, 1704-GDGSG-1708) and an N-2′-*O*-MTase motif, namely K–D–K–E catalytic tetrad (K1685–D1797–K1829–E1867) [106]. Recombinant RABV expressing the L protein with a mutation in the 2′-*O*-MTase motif (e.g., K1685A, K1829A) is severely attenuated and more sensitive to IFIT2, an IFIT1-related anti-viral protein, than wild-type virus [152]. Given these observations, the putative RABV MTase domain can be suggested to methylate the cap structure of RABV mRNAs at the two positions (Figure 5B), rendering them more translatable and resistant to anti-viral factors in infected cells.

## 5. Roles of the Rabies Virus (RABV) GDP Polyribonucleotidyltransferase (PRNTase) Domain in RNA Biosynthesis

**Figure 6:**
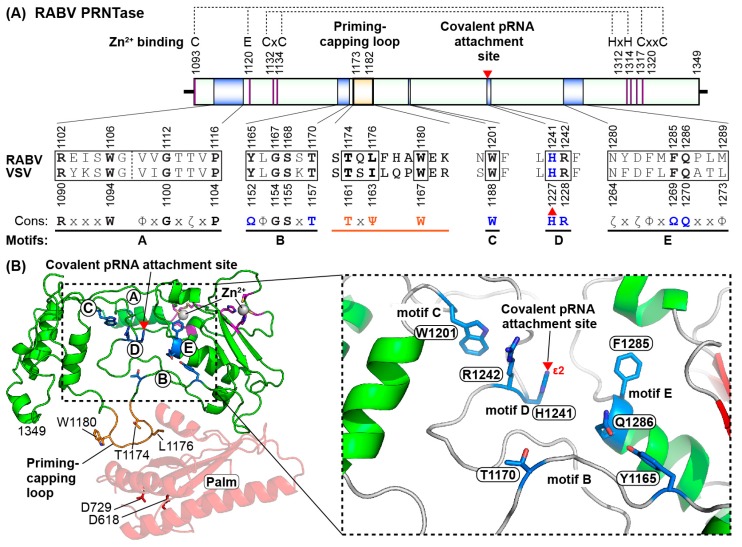
A predicted structure of the GDP polyribonucleotidyltransferase (PRNTase) domain in the rabies virus (RABV) large (L) protein. (**A**) A schematic structure of the RABV PRNTase domain is shown with amino acid sequences of the PRNTase motifs A–E and priming-capping loops of RABV and vesicular stomatitis virus (VSV). Consensus sequences of the PRNTase motifs among nonsegmented negative-strand RNA viruses and the priming-capping loops among animal/arthropod rhabdoviruses are indicated (Φ, hydrophobic amino acids; other symbols, see Figure 4). Motif D of the VSV PRNTase domain includes a nucleophilic histidine residue, which serves as a covalent pRNA attachment site (marked by a red arrowhead). The positions of amino acid residues involved in putative Zn^2+^-binding are shown by magenta lines in the schematic structure. (**B**) The modeled structures of the RABV PRNTase domain (green) and RNA-dependent RNA polymerase palm subdomain (pale red) are represented as ribbon models with key amino acid residues (stick models) (left). The positions of the PRNTase motifs A–E are shown by circled capital letters. Putative Zn^2+^-binding amino acid residues and two Zn^2+^ ions are shown as magenta stick models and silver spheres, respectively. The structural motif that coordinates the second Zn^2+^ (CxCx(177)HxH, residues C1132, C1134, H1312, H1314, top right) is different in RABV versus VSV (CxxCx(170)HxH, residues C1120, C1123, H1294, H1296). Moderate adjustments were made in the structural model to accommodate Zn^2+^-binding at this site. A close-up view of an active site of the PRNTase domain is shown on the right. The *N*^ε2^ position of H1241 in motif D may be covalently linked to pRNA for pre-mRNA capping. Adjusted coordinates of the modeled RABV L protein have been uploaded to the Model Archive (id: ma-pmodq).

The putative RABV PRNTase domain (residues 1093–1349) shares five conserved motifs, Rx(3)Wx(3–8)ΦxGxζx(P/A) (motif A), (Y/W)ΦGSxT (motif B), W (motif C), HR (motif D), and ζxxΦx(F/Y)QxxΦ (motif E) (Φ, hydrophobic; ζ, hydrophilic amino acids) with those in L proteins of NNS RNA viruses belonging to the the order *Mononegavirales* [8,107,144,153] (Figure 6A). In the flat PRNTase domain of the VSV L protein (PDB id: 5A22) [108], motifs B–E compose a unique active site with a putative substrate binding cavity, whereas motif A seems to provide a platform for the active site organization [8,153]. The putative RABV PRNTase domain was predicted to fold into a VSV PRNTase-like structure with a putative active site surrounded by motifs B–E (Figure 6B). As reported for the VSV L protein [153], G1112 in motif A, T1170 in motif B, W1201 in motif C, H1241 and R1242 in motif D, and F1285 and Q1286 in motif E were identified as essential for the PRNTase activity of the RABV L protein [46]. Similar mutations in the VSV PRNTase motifs are lethal to VSV [153,154]. Mass spectrometric and biochemical analyses of the VSV L–pRNA intermediate revealed that the *N*^ε2^ position of H1227 in motif D is covalently linked to the 5′-monophosphate end of the RNA with the VSV mRNA-start sequence via a phosphoamide bond [145]. Therefore, the RABV counterpart (H1241) of the VSV H1227 residue can be predicted to serve as a covalent pRNA attachment site for the putative L–pRNA intermediate formation. The nucleophilic histidine residue in motif D may attack the α-phosphorus in the 5′-triphosphate group of the pRNA donor, resulting in the formation of the L–pRNA intermediate with concomitant release of inorganic pyrophosphate (PP_i_). Roles of other key residues in motifs B–E in interactions with the substrates and products in the two-step ping-pong capping reaction were recently predicted based on an in silico docking study [8], but await experimental verification. 

The structural model of the putative RABV PRNTase domain suggests that it possesses a large loop structure flanking the PRNTase motif B (Figure 6B), which corresponds to a “priming loop” proposed for the VSV L protein [108]. De novo initiating RdRps of viruses (e.g., bacteriophage Φ6, reovirus, dengue virus, hepatitis C virus, influenza virus) often have a priming loop, which facilitates primer-independent initiation of RNA synthesis form 3′-termini of viral genomes by stabilizing their initiation complexes with initiator and incoming nucleotides [155,156,157,158,159]. As reported in the structure of the VSV L protein [108], the putative RABV priming loop was predicted to extend from the PRNTase domain toward the active site center of the RABV RdRp domain. To analyze the roles of the loop structure of RABV as well as VSV, effects of mutations in the loop on RNA synthesis and capping were examined [21]. The results showed that a TxΨ (Ψ, aliphatic amino acids) motif (T1174-x-L1176 for RABV, T1161-x-I1163 for VSV) on the loop is required for RNA capping, whereas a conserved tryptophan (W) residue (W1180 for RABV, W1167 for VSV) is essential for terminal de novo initiation from position 1 of the *Le*(−) promoter (3′-HO-U_1_G_2_-) to carry out the first phosphodiester bond formation (synthesis of pppAC) in a template-dependent manner [21]. In contrast, the TxΨ motif and W residue are dispensable for transcription initiation and capping, respectively [21]. These findings indicate that the putative loop structure extended from the PRNTase domain, named “priming-capping loop”, plays dual roles in transcription initiation and mRNA capping. Both the W residue and TxΨ motif are conserved in L proteins of rhabdoviruses infecting animals and/or arthropods, but not in those of other NNS RNA viruses, indicating their specific functions in these rhabdoviruses [8,21].

**Figure 7:**
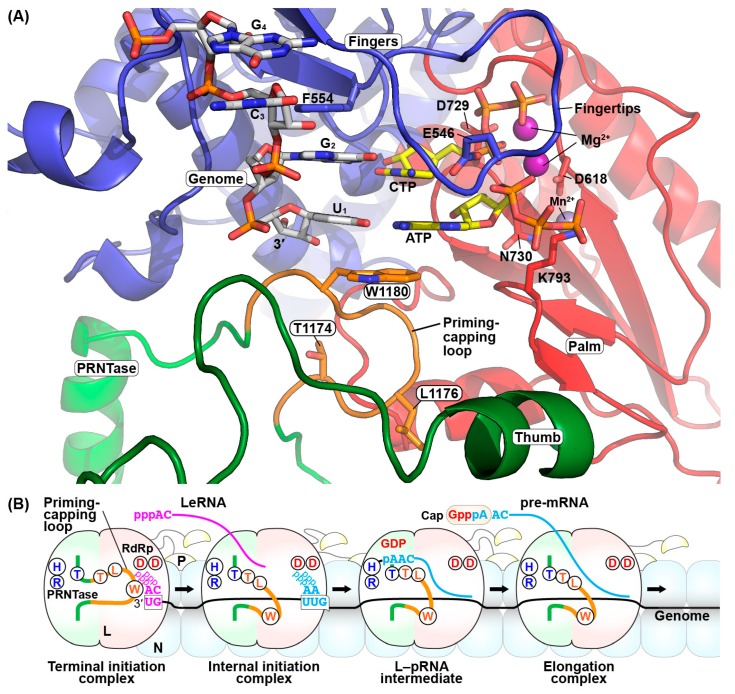
The roles of the GDP polyribonucleotidyltransferase (PRNTase) domain of the rabies virus (RABV) large (L) protein in transcription initiation and pre-mRNA capping. (**A**) The structure of an RABV terminal de novo initiation complex with a model RNA template (3′-UGCG-5′, white carbon backbone), initiator and incoming nucleotides (ATP and CTP, respectively; yellow carbon backbone), and divalent metal ions (Mg^2+^, purple; Mn^2+^, obscured) was modeled from the vesicular stomatitis virus initiation complex (Molecular Archive id: ma-5k432) based on the structure in [21]. The RNA-dependent RNA polymerase (RdRp) subdomains and PRNTase/priming loop of the RABV L protein are colored as in Figure 4. Key amino acid residues are shown as stick models. The coordinates of the modeled RABV terminal de novo initiation complex have been uploaded to the Model Archive (id: ma-uqibu). (**B**) A model for de novo transcription initiation and pre-mRNA capping by the RABV L protein is schematically presented. Only the PRNTase (pale green) and RdRp (pale red) domains of the L protein are depicted with the priming-capping loop (orange). Circled capital letters indicate the following amino acid residues: “T”, “H”, and “R” in the PRNTase domain are T1170, H1241, and R1242, respectively; “T”, “L”, and “W” on the priming-capping loop are T1174, L1176, and W1180, respectively; left and right “D”s in the RdRp domain are D618 and D729, respectively. The 3′-terminal UG sequence of the *Le*(−) promoter and the internal UUG sequence of the *N* gene-start sequence are shown on the genome (black thick line). pppA and pppC indicate ATP and CTP, respectively. The nucleocapsid (N) and phospho- (P) proteins are shown in pale blue and pale yellow, respectively.

Based on the structures of the bacteriophage Φ6 initiation complex (PDB id: 1HI0) [155] and the apo form of the VSV L protein (PDB id: 5A22) [108], we previously modeled the VSV initiation complex [21]. Using the VSV complex (Model Archive id: ma-5k432) as a template, we modeled the RABV initiation complex with the RABV RNA template (3′-U_1_G_2_C_3_G_4_), initiator ATP, incoming CTP, and divalent cations (2 Mg^2+^ and 1 Mn^2+^) (Figure 7A) with the secondary structure matching (SSM) tool in COOT [160] and energy minimization in PHENIX [161]. Similar to the VSV model, ATP and CTP were predicted to be base-paired with the U_1_ and G_2_ residues of the RABV model template, respectively, and are located adjacent to the active site aspartate residues (D618 and D729 in motifs A and C, respectively) in the palm subdomain of the RABV RdRp domain. The D729 residue was suggested to be associated with the α-phosphate of CTP sitting adjacent to a coordinated Mg^2+^ ion. This model also suggests that the E546 and R552 residues in the putative fingertips structure (motif F) interacts with the *C*^4^-amino group and α-phosphate, respectively, of CTP, and the phenyl group of the F554 residue sits stacked in-line with the U_1_ and G_2_ bases of the model template. Furthermore, the adenine base of ATP was predicted to be sandwiched between the cytosine base of CTP and the indole group of the W1180 residue on the priming-capping loop via π-stacking interactions. All these putative interactions appear to be critical for the formation of the terminal de novo initiation complex of RABV. 

On the other hand, the W1180 residue on the priming-capping loop extended from the PRNTase domain of the RABV L protein is dispensable for internal initiation from the *N* gene-start sequence as well as a gene-start-like sequence present in the *Le*(−) promoter (see Figure 1) [21]. It is intriguing to note that a proline residue on the priming loop extended from the thumb subdomain of the influenza A virus RdRp is required for terminal initiation from the genomic promoter, but not for internal initiation from the anti-genomic promoter [159]. Therefore, it is apparent that the mechanism of internal initiation is different from that of priming loop-dependent terminal initiation by these negative-strand RNA viral RdRps. Using an in vitro transcription system with oligo-RNAs as templates, the RABV RdRp was shown to use an internal 3′-A_-1_/U_+1_UGUNG-5′ sequence as an internal initiation sequence [21]. The internal initiation signals in the RABV gene-start-sequences are employed to produce 5′-pppAAC-strated pre-mRNA, which is subsequently capped with the PRNTase domain of the RABV L protein [46]. However, it is currently not known whether the gene-start-like sequence in the *Le*(−) promoter serves as an internal initiation signal to synthesize a 5′-pppAAC-strated RNA(s) in infected cells. 

Our biochemical data combined with the structural models generated here and in the recent studies [21] suggest that the priming-capping loop of the PRNTase domain in rhabdoviral L proteins performs the dual-functions in the sequential stop-start transcription (Figure 7B). In the step of terminal de novo initiation for LeRNA synthesis, the conserved W residue on the priming-capping loop stabilizes the RdRp complex with ATP and CTP at the 3′-terminal UG sequence of the genome to mediate the first phosphodiester bond formation (pppApC synthesis). To elongate and release LeRNA, the priming-capping loop may be retracted from the active site cavity of the RdRp domain. In the step of internal de novo initiation for mRNA synthesis at the gene-start sequence, the W residue on the priming-capping loop is no longer required for the first phosphodiester bond formation (pppApA synthesis). The TxΨ motif on the priming-capping loop of RABV as well as VSV is critical for capping of 5′-pppApApC-started RNAs. For VSV, the TxΨ motif was shown to be essential for the L–pRNA intermediate formation with pppAACAG, the VSV mRNA-start sequence [21]. These observations suggest that a conformational change in the priming-capping loop may bring the TxΨ motif close to the PRNTase active site, leading to its distinct configuration to recognize the rhabdovirus specific mRNA-start sequence for pre-mRNA capping. If the VSV L protein has a mutation abolishing the L–pRNA intermediate formation in the TxΨ motif as well as the PRNTase motifs, it frequently terminates and reinitiates transcription using suboptimal termination and initiation signals within the *N* gene, producing unusual 5′-triphosphorylated *N* mRNA fragments [21,153,154]. Therefore, the L–pRNA intermediate formation mediated by the priming-capping loop during mRNA chain elongation is a key step leading to progression of the downstream events, such as the pRNA transfer to form the 5′-cap structure, continuous elongation, accurate 3′-polyadenylation and termination at the gene-end sequence, and eventually release of mature mRNA.

## 6. Concluding Remarks

Studies on RNA synthesis and processing with the RABV RdRp have been limited by the lack of efficient in vitro transcription systems over the past four decades. As described in this review, the recent development of the in vitro RNA synthesis and capping systems with the recombinant RABV L protein allowed us to reveal the enzymatic and regulatory roles of the L protein in transcription initiation and capping. The enzymatically active recombinant RABV L protein may open up new opportunities to elucidate roles of the putative MTase domain in cap methylation and to locate the binding site for the co-factor P protein. Further elucidation of the molecular mechanisms of mRNA and genome/antigenome biosynthesis by the RABV L protein and its structural analyses would help in the development of antiviral agents targeting its essential domains.

## References

[1] Albertini A.A., Ruigrok R.W., Blondel D. (2011). Rabies virus transcription and replication. Adv. Virus Res..

[2] Davis B.M., Rall G.F., Schnell M.J. (2015). Everything you always wanted to know about rabies virus (but were afraid to ask). Annu. Rev. Virol..

[3] Fisher C.R., Streicker D.G., Schnell M.J. (2018). The spread and evolution of rabies virus: Conquering new frontiers. Nat. Rev. Microbiol..

[4] Du Pont V., Plemper R.K., Schnell M.J. (2019). Status of antiviral therapeutics against rabies virus and related emerging lyssaviruses. Curr. Opin. Virol..

[5] Fooks A.R., Banyard A.C., Horton D.L., Johnson N., McElhinney L.M., Jackson A.C. (2014). Current status of rabies and prospects for elimination. Lancet.

[6] Lankester F., Hampson K., Lembo T., Palmer G., Taylor L., Cleaveland S. (2014). Infectious Disease. Implementing Pasteur’s vision for rabies elimination. Science.

[7] Johnson N., Vos A., Freuling C., Tordo N., Fooks A.R., Muller T. (2010). Human rabies due to lyssavirus infection of bat origin. Vet. Microbiol..

[8] Ogino T., Green T.J. (2019). RNA synthesis and capping by non-segmented negative strand RNA viral polymerases: Lessons from a prototypic virus. Front. Microbiol..

[9] Villarreal L.P., Holland J.J. (1974). Transcribing complexes in cells infected by vesicular stomatitis virus and rabies virus. J. Virol..

[10] Kawai A. (1977). Transcriptase activity associated with rabies virion. J. Virol..

[11] Flamand A., Delagneau J.F., Bussereau F. (1978). An RNA polymerase activity in purified rabies virions. J. Gen. Virol..

[12] Tordo N., Poch O., Ermine A., Keith G. (1986). Primary structure of leader RNA and nucleoprotein genes of the rabies genome: Segmented homology with VSV. Nucleic Acids Res..

[13] Tordo N., Poch O., Ermine A., Keith G., Rougeon F. (1986). Walking along the rabies genome: Is the large G-L intergenic region a remnant gene?. Proc. Natl. Acad. Sci. USA.

[14] Tordo N., Poch O., Ermine A., Keith G., Rougeon F. (1988). Completion of the rabies virus genome sequence determination: Highly conserved domains among the L (polymerase) proteins of unsegmented negative-strand RNA viruses. Virology.

[15] Conzelmann K.K., Cox J.H., Schneider L.G., Thiel H.J. (1990). Molecular cloning and complete nucleotide sequence of the attenuated rabies virus SAD B19. Virology.

[16] Bourhy H., Kissi B., Tordo N. (1993). Molecular diversity of the Lyssavirus genus. Virology.

[17] Delmas O., Holmes E.C., Talbi C., Larrous F., Dacheux L., Bouchier C., Bourhy H. (2008). Genomic diversity and evolution of the lyssaviruses. PLoS ONE.

[18] Conzelmann K.K., Schnell M. (1994). Rescue of synthetic genomic RNA analogs of rabies virus by plasmid-encoded proteins. J. Virol..

[19] Schnell M.J., Mebatsion T., Conzelmann K.K. (1994). Infectious rabies viruses from cloned cDNA. EMBO J..

[20] Finke S., Conzelmann K.K. (1997). Ambisense gene expression from recombinant rabies virus: Random packaging of positive- and negative-strand ribonucleoprotein complexes into rabies virions. J. Virol..

[21] Ogino M., Gupta N., Green T.J., Ogino T. (2019). A dual-functional priming-capping loop of rhabdoviral RNA polymerases directs terminal de novo initiation and capping intermediate formation. Nucleic Acids Res..

[22] Barr J.N., Whelan S.P., Wertz G.W. (1997). cis-Acting signals involved in termination of vesicular stomatitis virus mRNA synthesis include the conserved AUAC and the U7 signal for polyadenylation. J. Virol..

[23] Stillman E.A., Whitt M.A. (1999). Transcript initiation and 5′-end modifications are separable events during vesicular stomatitis virus transcription. J. Virol..

[24] Iseni F., Barge A., Baudin F., Blondel D., Ruigrok R.W. (1998). Characterization of rabies virus nucleocapsids and recombinant nucleocapsid-like structures. J. Gen. Virol..

[25] Emerson S.U., Wagner R.R. (1972). Dissociation and reconstitution of the transcriptase and template activities of vesicular stomatitis B and T virions. J. Virol..

[26] Emerson S.U., Yu Y. (1975). Both NS and L proteins are required for in vitro RNA synthesis by vesicular stomatitis virus. J. Virol..

[27] Mellon M.G., Emerson S.U. (1978). Rebinding of transcriptase components (L and NS proteins) to the nucleocapsid template of vesicular stomatitis virus. J. Virol..

[28] De B.P., Banerjee A.K. (1984). Specific interactions of vesicular stomatitis virus L and NS proteins with heterologous genome ribonucleoprotein template lead to mRNA synthesis in vitro. J. Virol..

[29] De B.P., Banerjee A.K. (1985). Requirements and functions of vesicular stomatitis virus L and NS proteins in the transcription process in vitro. Biochem. Biophys. Res. Commun..

[30] Chenik M., Schnell M., Conzelmann K.K., Blondel D. (1998). Mapping the interacting domains between the rabies virus polymerase and phosphoprotein. J. Virol..

[31] Schoehn G., Iseni F., Mavrakis M., Blondel D., Ruigrok R.W. (2001). Structure of recombinant rabies virus nucleoprotein-RNA complex and identification of the phosphoprotein binding site. J. Virol..

[32] Mavrakis M., McCarthy A.A., Roche S., Blondel D., Ruigrok R.W. (2004). Structure and function of the C-terminal domain of the polymerase cofactor of rabies virus. J. Mol. Biol..

[33] Ribeiro Ede A., Leyrat C., Gerard F.C., Albertini A.A., Falk C., Ruigrok R.W., Jamin M. (2009). Binding of rabies virus polymerase cofactor to recombinant circular nucleoprotein-RNA complexes. J. Mol. Biol..

[34] Castel G., Chteoui M., Caignard G., Prehaud C., Mehouas S., Real E., Jallet C., Jacob Y., Ruigrok R.W., Tordo N. (2009). Peptides that mimic the amino-terminal end of the rabies virus phosphoprotein have antiviral activity. J. Virol..

[35] Lahaye X., Vidy A., Pomier C., Obiang L., Harper F., Gaudin Y., Blondel D. (2009). Functional characterization of Negri bodies (NBs) in rabies virus-infected cells: Evidence that NBs are sites of viral transcription and replication. J. Virol..

[36] Nikolic J., Civas A., Lama Z., Lagaudriere-Gesbert C., Blondel D. (2016). Rabies virus infection induces the formation of stress granules closely connected to the viral factories. PLoS Pathog..

[37] Nikolic J., Le Bars R., Lama Z., Scrima N., Lagaudriere-Gesbert C., Gaudin Y., Blondel D. (2017). Negri bodies are viral factories with properties of liquid organelles. Nat. Commun..

[38] Heinrich B.S., Maliga Z., Stein D.A., Hyman A.A., Whelan S.P.J. (2018). Phase transitions drive the formation of vesicular stomatitis virus replication compartments. MBio.

[39] Crooks G.E., Hon G., Chandonia J.M., Brenner S.E. (2004). WebLogo: A sequence logo generator. Genome Res..

[40] Smallwood S., Moyer S.A. (1993). Promoter analysis of the vesicular stomatitis virus RNA polymerase. Virology.

[41] Barr J.N., Whelan S.P., Wertz G.W. (1997). Role of the intergenic dinucleotide in vesicular stomatitis virus RNA transcription. J. Virol..

[42] Stillman E.A., Whitt M.A. (1998). The length and sequence composition of vesicular stomatitis virus intergenic regions affect mRNA levels and the site of transcript initiation. J. Virol..

[43] Ogino T., Banerjee A.K. (2007). Unconventional mechanism of mRNA capping by the RNA-dependent RNA polymerase of vesicular stomatitis virus. Mol. Cell.

[44] Ogino T., Banerjee A.K. (2008). Formation of guanosine(5′)tetraphospho(5′)adenosine cap structure by an unconventional mRNA capping enzyme of vesicular stomatitis virus. J. Virol..

[45] Morin B., Rahmeh A.A., Whelan S.P. (2012). Mechanism of RNA synthesis initiation by the vesicular stomatitis virus polymerase. EMBO J..

[46] Ogino M., Ito N., Sugiyama M., Ogino T. (2016). The rabies virus L protein catalyzes mRNA capping with GDP polyribonucleotidyltransferase activity. Viruses.

[47] Abraham G., Rhodes D.P., Banerjee A.K. (1975). The 5′ terminal structure of the methylated mRNA synthesized in vitro by vesicular stomatitis virus. Cell.

[48] Abraham G., Banerjee A.K. (1976). Sequential transcription of the genes of vesicular stomatitis virus. Proc. Natl. Acad. Sci. USA.

[49] Ball L.A., White C.N. (1976). Order of transcription of genes of vesicular stomatitis virus. Proc. Natl. Acad. Sci. USA.

[50] Testa D., Chanda P.K., Banerjee A.K. (1980). Unique mode of transcription in vitro by vesicular stomatitis virus. Cell.

[51] Emerson S.U. (1982). Reconstitution studies detect a single polymerase entry site on the vesicular stomatitis virus genome. Cell.

[52] Flamand A., Delagneau J.F. (1978). Transcriptional mapping of rabies virus in vivo. J. Virol..

[53] Coslett G.D., Holloway B.P., Obijeski J.F. (1980). The structural proteins of rabies virus and evidence for their synthesis from separate monocistronic RNA species. J. Gen. Virol..

[54] Holloway B.P., Obijeski J.F. (1980). Rabies virus-induced RNA synthesis in BHK21 cells. J. Gen. Virol..

[55] Moyer S.A., Abraham G., Adler R., Banerjee A.K. (1975). Methylated and blocked 5′ termini in vesicular stomatitis virus in vivo mRNAs. Cell.

[56] Moyer S.A., Banerjee A.K. (1976). In vivo methylation of vesicular stomatitis virus and its host-cell messenger RNA species. Virology.

[57] Kurilla M.G., Cabradilla C.D., Holloway B.P., Keene J.D. (1984). Nucleotide sequence and host La protein interactions of rabies virus leader RNA. J. Virol..

[58] Iverson L.E., Rose J.K. (1981). Localized attenuation and discontinuous synthesis during vesicular stomatitis virus transcription. Cell.

[59] Finke S., Cox J.H., Conzelmann K.K. (2000). Differential transcription attenuation of rabies virus genes by intergenic regions: Generation of recombinant viruses overexpressing the polymerase gene. J. Virol..

[60] Rose J.K. (1980). Complete intergenic and flanking gene sequences from the genome of vesicular stomatitis virus. Cell.

[61] Peluso R.W. (1988). Kinetic, quantitative, and functional analysis of multiple forms of the vesicular stomatitis virus nucleocapsid protein in infected cells. J. Virol..

[62] Peluso R.W., Moyer S.A. (1988). Viral proteins required for the in vitro replication of vesicular stomatitis virus defective interfering particle genome RNA. Virology.

[63] Masters P.S., Banerjee A.K. (1988). Complex formation with vesicular stomatitis virus phosphoprotein NS prevents binding of nucleocapsid protein N to nonspecific RNA. J. Virol..

[64] Gupta A.K., Banerjee A.K. (1997). Expression and purification of vesicular stomatitis virus N-P complex from Escherichia coli: Role in genome RNA transcription and replication in vitro. J. Virol..

[65] Mavrakis M., Iseni F., Mazza C., Schoehn G., Ebel C., Gentzel M., Franz T., Ruigrok R.W. (2003). Isolation and characterisation of the rabies virus N degrees-P complex produced in insect cells. Virology.

[66] Blumberg B.M., Giorgi C., Kolakofsky D. (1983). N protein of vesicular stomatitis virus selectively encapsidates leader RNA in vitro. Cell.

[67] Blumberg B.M., Leppert M., Kolakofsky D. (1981). Interaction of VSV leader RNA and nucleocapsid protein may control VSV genome replication. Cell.

[68] Yang J., Hooper D.C., Wunner W.H., Koprowski H., Dietzschold B., Fu Z.F. (1998). The specificity of rabies virus RNA encapsidation by nucleoprotein. Virology.

[69] Finke S., Conzelmann K.K. (2003). Dissociation of rabies virus matrix protein functions in regulation of viral RNA synthesis and virus assembly. J. Virol..

[70] Finke S., Mueller-Waldeck R., Conzelmann K.K. (2003). Rabies virus matrix protein regulates the balance of virus transcription and replication. J. Gen. Virol..

[71] Green T.J., Macpherson S., Qiu S., Lebowitz J., Wertz G.W., Luo M. (2000). Study of the assembly of vesicular stomatitis virus N protein: Role of the P protein. J. Virol..

[72] Green T.J., Zhang X., Wertz G.W., Luo M. (2006). Structure of the vesicular stomatitis virus nucleoprotein-RNA complex. Science.

[73] Albertini A.A., Wernimont A.K., Muziol T., Ravelli R.B., Clapier C.R., Schoehn G., Weissenhorn W., Ruigrok R.W. (2006). Crystal structure of the rabies virus nucleoprotein-RNA complex. Science.

[74] Zhang X., Green T.J., Tsao J., Qiu S., Luo M. (2008). Role of intermolecular interactions of vesicular stomatitis virus nucleoprotein in RNA encapsidation. J. Virol..

[75] Rainsford E.W., Harouaka D., Wertz G.W. (2010). Importance of hydrogen bond contacts between the N protein and RNA genome of vesicular stomatitis virus in encapsidation and RNA synthesis. J. Virol..

[76] Green T.J., Luo M. (2009). Structure of the vesicular stomatitis virus nucleocapsid in complex with the nucleocapsid-binding domain of the small polymerase cofactor, P. Proc. Natl. Acad. Sci. USA.

[77] Wu X., Gong X., Foley H.D., Schnell M.J., Fu Z.F. (2002). Both viral transcription and replication are reduced when the rabies virus nucleoprotein is not phosphorylated. J. Virol..

[78] Leyrat C., Yabukarski F., Tarbouriech N., Ribeiro E.A., Jensen M.R., Blackledge M., Ruigrok R.W., Jamin M. (2011). Structure of the vesicular stomatitis virus N(0)-P complex. PLoS Pathog..

[79] Gupta A.K., Blondel D., Choudhary S., Banerjee A.K. (2000). The phosphoprotein of rabies virus is phosphorylated by a unique cellular protein kinase and specific isomers of protein kinase C. J. Virol..

[80] Gerard F.C., Ribeiro Ede A., Albertini A.A., Gutsche I., Zaccai G., Ruigrok R.W., Jamin M. (2007). Unphosphorylated rhabdoviridae phosphoproteins form elongated dimers in solution. Biochemistry.

[81] Gerard F.C., Ribeiro Ede A., Leyrat C., Ivanov I., Blondel D., Longhi S., Ruigrok R.W., Jamin M. (2009). Modular organization of rabies virus phosphoprotein. J. Mol. Biol..

[82] Mavrakis M., Mehouas S., Real E., Iseni F., Blondel D., Tordo N., Ruigrok R.W. (2006). Rabies virus chaperone: Identification of the phosphoprotein peptide that keeps nucleoprotein soluble and free from non-specific RNA. Virology.

[83] Morin B., Liang B., Gardner E., Ross R.A., Whelan S.P. (2017). An in vitro RNA synthesis assay for rabies virus defines ribonucleoprotein interactions critical for polymerase activity. J. Virol..

[84] Ivanov I., Crepin T., Jamin M., Ruigrok R.W. (2010). Structure of the dimerization domain of the rabies virus phosphoprotein. J. Virol..

[85] Jacob Y., Real E., Tordo N. (2001). Functional interaction map of lyssavirus phosphoprotein: Identification of the minimal transcription domains. J. Virol..

[86] Jacob Y., Badrane H., Ceccaldi P.E., Tordo N. (2000). Cytoplasmic dynein LC8 interacts with lyssavirus phosphoprotein. J. Virol..

[87] Raux H., Flamand A., Blondel D. (2000). Interaction of the rabies virus P protein with the LC8 dynein light chain. J. Virol..

[88] Poisson N., Real E., Gaudin Y., Vaney M.C., King S., Jacob Y., Tordo N., Blondel D. (2001). Molecular basis for the interaction between rabies virus phosphoprotein P and the dynein light chain LC8: Dissociation of dynein-binding properties and transcriptional functionality of P. J. Gen. Virol..

[89] Tan G.S., Preuss M.A., Williams J.C., Schnell M.J. (2007). The dynein light chain 8 binding motif of rabies virus phosphoprotein promotes efficient viral transcription. Proc. Natl. Acad. Sci. USA.

[90] Fouquet B., Nikolic J., Larrous F., Bourhy H., Wirblich C., Lagaudriere-Gesbert C., Blondel D. (2015). Focal adhesion kinase is involved in rabies virus infection through its interaction with viral phosphoprotein P. J. Virol..

[91] Rieder M., Conzelmann K.K. (2011). Interferon in rabies virus infection. Adv. Virus Res..

[92] Rieder M., Brzozka K., Pfaller C.K., Cox J.H., Stitz L., Conzelmann K.K. (2011). Genetic dissection of interferon-antagonistic functions of rabies virus phosphoprotein: Inhibition of interferon regulatory factor 3 activation is important for pathogenicity. J. Virol..

[93] Vidy A., Chelbi-Alix M., Blondel D. (2005). Rabies virus P protein interacts with STAT1 and inhibits interferon signal transduction pathways. J. Virol..

[94] Brzozka K., Finke S., Conzelmann K.K. (2006). Inhibition of interferon signaling by rabies virus phosphoprotein P: Activation-dependent binding of STAT1 and STAT2. J. Virol..

[95] Blondel D., Regad T., Poisson N., Pavie B., Harper F., Pandolfi P.P., De The H., Chelbi-Alix M.K. (2002). Rabies virus P and small P products interact directly with PML and reorganize PML nuclear bodies. Oncogene.

[96] Hunt D.M., Hutchinson K.L. (1993). Amino acid changes in the L polymerase protein of vesicular stomatitis virus which confer aberrant polyadenylation and temperature-sensitive phenotypes. Virology.

[97] Grdzelishvili V.Z., Smallwood S., Tower D., Hall R.L., Hunt D.M., Moyer S.A. (2005). A single amino acid change in the L-polymerase protein of vesicular stomatitis virus completely abolishes viral mRNA cap methylation. J. Virol..

[98] Li J., Fontaine-Rodriguez E.C., Whelan S.P. (2005). Amino acid residues within conserved domain VI of the vesicular stomatitis virus large polymerase protein essential for mRNA cap methyltransferase activity. J. Virol..

[99] Ogino T., Kobayashi M., Iwama M., Mizumoto K. (2005). Sendai virus RNA-dependent RNA polymerase L protein catalyzes cap methylation of virus-specific mRNA. J. Biol. Chem..

[100] Galloway S.E., Wertz G.W. (2008). S-adenosyl homocysteine-induced hyperpolyadenylation of vesicular stomatitis virus mRNA requires the methyltransferase activity of L protein. J. Virol..

[101] Rahmeh A.A., Li J., Kranzusch P.J., Whelan S.P. (2009). Ribose 2′-O methylation of the vesicular stomatitis virus mRNA cap precedes and facilitates subsequent guanine-N-7 methylation by the large polymerase protein. J. Virol..

[102] Paesen G.C., Collet A., Sallamand C., Debart F., Vasseur J.J., Canard B., Decroly E., Grimes J.M. (2015). X-ray structure and activities of an essential Mononegavirales L-protein domain. Nat. Commun..

[103] Jordan P.C., Liu C., Raynaud P., Lo M.K., Spiropoulou C.F., Symons J.A., Beigelman L., Deval J. (2018). Initiation, extension, and termination of RNA synthesis by a paramyxovirus polymerase. PLoS Pathog..

[104] Nakagawa K., Kobayashi Y., Ito N., Suzuki Y., Okada K., Makino M., Goto H., Takahashi T., Sugiyama M. (2017). Molecular function analysis of rabies virus rna polymerase l protein by using an l gene-deficient virus. J. Virol..

[105] Poch O., Blumberg B.M., Bougueleret L., Tordo N. (1990). Sequence comparison of five polymerases (L proteins) of unsegmented negative-strand RNA viruses: Theoretical assignment of functional domains. J. Gen. Virol..

[106] Bujnicki J.M., Rychlewski L. (2002). In silico identification, structure prediction and phylogenetic analysis of the 2′-O-ribose (cap 1) methyltransferase domain in the large structural protein of ssRNA negative-strand viruses. Protein Eng..

[107] Ogino T., Banerjee A.K. (2011). An unconventional pathway of mRNA cap formation by vesiculoviruses. Virus Res..

[108] Liang B., Li Z., Jenni S., Rahmeh A.A., Morin B.M., Grant T., Grigorieff N., Harrison S.C., Whelan S.P. (2015). Structure of the L protein of vesicular stomatitis virus from electron cryomicroscopy. Cell.

[109] Qiu S., Ogino M., Luo M., Ogino T., Green T.J. (2016). Structure and function of the N-terminal domain of the vesicular stomatitis virus RNA polymerase. J. Virol..

[110] O’Reilly E.K., Kao C.C. (1998). Analysis of RNA-dependent RNA polymerase structure and function as guided by known polymerase structures and computer predictions of secondary structure. Virology.

[111] Bruenn J.A. (2003). A structural and primary sequence comparison of the viral RNA-dependent RNA polymerases. Nucleic Acids Res..

[112] Lang D.M., Zemla A.T., Zhou C.L. (2013). Highly similar structural frames link the template tunnel and NTP entry tunnel to the exterior surface in RNA-dependent RNA polymerases. Nucleic Acids Res..

[113] Steitz T.A. (1998). A mechanism for all polymerases. Nature.

[114] Florian J., Goodman M.F., Warshel A. (2003). Computer simulation of the chemical catalysis of DNA polymerases: Discriminating between alternative nucleotide insertion mechanisms for T7 DNA polymerase. J. Am. Chem. Soc..

[115] Florian J., Goodman M.F., Warshel A. (2005). Computer simulations of protein functions: Searching for the molecular origin of the replication fidelity of DNA polymerases. Proc. Natl. Acad. Sci. USA.

[116] Castro C., Smidansky E., Maksimchuk K.R., Arnold J.J., Korneeva V.S., Gotte M., Konigsberg W., Cameron C.E. (2007). Two proton transfers in the transition state for nucleotidyl transfer catalyzed by RNA- and DNA-dependent RNA and DNA polymerases. Proc. Natl. Acad. Sci. USA.

[117] Castro C., Smidansky E.D., Arnold J.J., Maksimchuk K.R., Moustafa I., Uchida A., Gotte M., Konigsberg W., Cameron C.E. (2009). Nucleic acid polymerases use a general acid for nucleotidyl transfer. Nat. Struct. Mol. Biol..

[118] Gong P., Peersen O.B. (2010). Structural basis for active site closure by the poliovirus RNA-dependent RNA polymerase. Proc. Natl. Acad. Sci. USA.

[119] Genna V., Vidossich P., Ippoliti E., Carloni P., de Vivo M. (2016). A self-activated mechanism for nucleic acid polymerization catalyzed by DNA/RNA polymerases. J. Am. Chem. Soc..

[120] Schnell M.J., Conzelmann K.K. (1995). Polymerase activity of in vitro mutated rabies virus L protein. Virology.

[121] Bauer A., Nolden T., Nemitz S., Perlson E., Finke S. (2015). A dynein light chain 1 binding motif in rabies virus polymerase l protein plays a role in microtubule reorganization and viral primary transcription. J. Virol..

[122] Waterhouse A., Bertoni M., Bienert S., Studer G., Tauriello G., Gumienny R., Heer F.T., de Beer T.A.P., Rempfer C., Bordoli L. (2018). SWISS-MODEL: Homology modelling of protein structures and complexes. Nucleic Acids Res..

[123] DeLano W.L. The PyMOL molecular graphics system. http://www.pymol.org/.

[124] Banerjee A.K. (1980). 5′-terminal cap structure in eucaryotic messenger ribonucleic acids. Microbiol. Rev..

[125] Furuichi Y., Shatkin A.J. (2000). Viral and cellular mRNA capping: Past and prospects. Adv. Virus Res..

[126] Shuman S. (2001). Structure, mechanism, and evolution of the mRNA capping apparatus. Prog. Nucleic Acid Res. Mol. Biol..

[127] Ghosh A., Lima C.D. (2010). Enzymology of RNA cap synthesis. Wiley Interdiscip. Rev. RNA.

[128] Ramanathan A., Robb G.B., Chan S.H. (2016). mRNA capping: Biological functions and applications. Nucleic Acids Res..

[129] Hyde J.L., Diamond M.S. (2015). Innate immune restriction and antagonism of viral RNA lacking 2-O methylation. Virology.

[130] Leung D.W., Amarasinghe G.K. (2016). When your cap matters: Structural insights into self vs non-self recognition of 5′ RNA by immunomodulatory host proteins. Curr. Opin. Struct. Biol..

[131] Schuberth-Wagner C., Ludwig J., Bruder A.K., Herzner A.M., Zillinger T., Goldeck M., Schmidt T., Schmid-Burgk J.L., Kerber R., Wolter S. (2015). A conserved histidine in the rna sensor RIG-I controls immune tolerance to N1-2′O-methylated self RNA. Immunity.

[132] Devarkar S.C., Wang C., Miller M.T., Ramanathan A., Jiang F., Khan A.G., Patel S.S., Marcotrigiano J. (2016). Structural basis for m7G recognition and 2′-O-methyl discrimination in capped RNAs by the innate immune receptor RIG-I. Proc. Natl. Acad. Sci. USA.

[133] Zust R., Cervantes-Barragan L., Habjan M., Maier R., Neuman B.W., Ziebuhr J., Szretter K.J., Baker S.C., Barchet W., Diamond M.S. (2011). Ribose 2′-O-methylation provides a molecular signature for the distinction of self and non-self mRNA dependent on the RNA sensor Mda5. Nat. Immunol..

[134] Daffis S., Szretter K.J., Schriewer J., Li J., Youn S., Errett J., Lin T.Y., Schneller S., Zust R., Dong H. (2010). 2′-O methylation of the viral mRNA cap evades host restriction by IFIT family members. Nature.

[135] Habjan M., Hubel P., Lacerda L., Benda C., Holze C., Eberl C.H., Mann A., Kindler E., Gil-Cruz C., Ziebuhr J. (2013). Sequestration by IFIT1 impairs translation of 2′O-unmethylated capped RNA. PLoS Pathog..

[136] Kumar P., Sweeney T.R., Skabkin M.A., Skabkina O.V., Hellen C.U., Pestova T.V. (2014). Inhibition of translation by IFIT family members is determined by their ability to interact selectively with the 5′-terminal regions of cap0-, cap1- and 5′ppp- mRNAs. Nucleic Acids Res..

[137] Daugherty M.D., Schaller A.M., Geballe A.P., Malik H.S. (2016). Evolution-guided functional analyses reveal diverse antiviral specificities encoded by IFIT1 genes in mammals. Elife.

[138] Abbas Y.M., Laudenbach B.T., Martinez-Montero S., Cencic R., Habjan M., Pichlmair A., Damha M.J., Pelletier J., Nagar B. (2017). Structure of human IFIT1 with capped RNA reveals adaptable mRNA binding and mechanisms for sensing N1 and N2 ribose 2′-O methylations. Proc. Natl. Acad. Sci. USA.

[139] Johnson B., VanBlargan L.A., Xu W., White J.P., Shan C., Shi P.Y., Zhang R., Adhikari J., Gross M.L., Leung D.W. (2018). Human IFIT3 modulates IFIT1 RNA binding specificity and protein stability. Immunity.

[140] Wei C., Gershowitz A., Moss B. (1975). N6, O2′-dimethyladenosine a novel methylated ribonucleoside next to the 5′ terminal of animal cell and virus mRNAs. Nature.

[141] Akichika S., Hirano S., Shichino Y., Suzuki T., Nishimasu H., Ishitani R., Sugita A., Hirose Y., Iwasaki S., Nureki O. (2019). Cap-specific terminal N (6)-methylation of RNA by an RNA polymerase II-associated methyltransferase. Science.

[142] Abraham G., Rhodes D.P., Banerjee A.K. (1975). Novel initiation of RNA synthesis in vitro by vesicular stomatitis virus. Nature.

[143] Ogino T., Banerjee A.K. (2010). The HR motif in the RNA-dependent RNA polymerase L protein of Chandipura virus is required for unconventional mRNA-capping activity. J. Gen. Virol..

[144] Ogino T., Banerjee A.K., Luo M. (2011). mRNA capping by vesicular stomatitis virus and other related viruses. Negative Strand RNA Virus.

[145] Ogino T., Yadav S.P., Banerjee A.K. (2010). Histidine-mediated RNA transfer to GDP for unique mRNA capping by vesicular stomatitis virus RNA polymerase. Proc. Natl. Acad. Sci. USA.

[146] Ogino T. (2013). In vitro capping and transcription of rhabdoviruses. Methods.

[147] Colonno R.J., Banerjee A.K. (1976). A unique RNA species involved in initiation of vesicular stomatitis virus RNA transcription in vitro. Cell.

[148] Ogino M., Ogino T. (2017). 5′-Phospho-RNA acceptor specificity of GDP polyribonucleotidyltransferase of vesicular stomatitis virus in mRNA capping. J. Virol..

[149] Testa D., Banerjee A.K. (1977). Two methyltransferase activities in the purified virions of vesicular stomatitis virus. J. Virol..

[150] Hercyk N., Horikami S.M., Moyer S.A. (1988). The vesicular stomatitis virus L protein possesses the mRNA methyltransferase activities. Virology.

[151] Li J., Wang J.T., Whelan S.P. (2006). A unique strategy for mRNA cap methylation used by vesicular stomatitis virus. Proc. Natl. Acad. Sci. USA.

[152] Tian D., Luo Z., Zhou M., Li M., Yu L., Wang C., Yuan J., Li F., Tian B., Sui B. (2016). Critical role of K1685 and K1829 in the large protein of rabies virus in viral pathogenicity and immune evasion. J. Virol..

[153] Neubauer J., Ogino M., Green T.J., Ogino T. (2016). Signature motifs of GDP polyribonucleotidyltransferase, a non-segmented negative strand RNA viral mRNA capping enzyme, domain in the L protein are required for covalent enzyme-pRNA intermediate formation. Nucleic Acids Res..

[154] Ogino T. (2014). Capping of vesicular stomatitis virus pre-mRNA is required for accurate selection of transcription stop-start sites and virus propagation. Nucleic Acids Res..

[155] Butcher S.J., Grimes J.M., Makeyev E.V., Bamford D.H., Stuart D.I. (2001). A mechanism for initiating RNA-dependent RNA polymerization. Nature.

[156] Tao Y., Farsetta D.L., Nibert M.L., Harrison S.C. (2002). RNA synthesis in a cage--structural studies of reovirus polymerase lambda3. Cell.

[157] Selisko B., Potisopon S., Agred R., Priet S., Varlet I., Thillier Y., Sallamand C., Debart F., Vasseur J.J., Canard B. (2012). Molecular basis for nucleotide conservation at the ends of the dengue virus genome. PLoS Pathog..

[158] Appleby T.C., Perry J.K., Murakami E., Barauskas O., Feng J., Cho A., Fox D., Wetmore D.R., McGrath M.E., Ray A.S. (2015). Viral replication. Structural basis for RNA replication by the hepatitis C virus polymerase. Science.

[159] Te Velthuis A.J., Robb N.C., Kapanidis A.N., Fodor E. (2016). The role of the priming loop in influenza A virus RNA synthesis. Nat. Microbiol..

[160] Emsley P., Lohkamp B., Scott W.G., Cowtan K. (2010). Features and development of Coot. Acta. Crystallogr. D Biol. Crystallogr..

[161] Adams P.D., Afonine P.V., Bunkoczi G., Chen V.B., Davis I.W., Echols N., Headd J.J., Hung L.W., Kapral G.J., Grosse-Kunstleve R.W. (2010). PHENIX: A comprehensive Python-based system for macromolecular structure solution. Acta. Crystallogr. D Biol. Crystallogr..

